# AI driven prediction of early age compressive strength in ultra high performance fiber reinforced concrete

**DOI:** 10.1038/s41598-025-06725-z

**Published:** 2025-06-26

**Authors:** Mohamed Abdellatief, Wafa Hamla, Hassan Hamouda

**Affiliations:** 1 Department of Civil Engineering, Higher Future Institute of Engineering and Technology in Mansoura, Mansoura, Egypt; 2 University Mohamed El Bachir El Ibrahimi of Bordj Bou Arreridj, Bordj Bou Arreridj, Algeria; 3 Civil and Architectural Construction Department, Faculty of Technology and Education, Suez University, Suez, Egypt

**Keywords:** UHPFRC, Early-age compressive strength, Gaussian process regression- feature importance analysis, Civil engineering, Nanoscale materials, Structural materials

## Abstract

Ultra-high-performance fiber-reinforced concrete (UHPFRC) is an exceptional type of cementitious composite with superior mechanical and durability performances. Achieving these properties involves maintaining a low water-to-cement ratio, optimizing aggregate size distribution, and integrating fiber reinforcement. Recently, there has been a notable trend in the development and application of UHPFRCs. However, there is still a requirement for artificial intelligence (AI) methods to predict the early-age compressive strength (CS) of UHPFRC and to define the key input factors for optimal mix design with appropriate proportions. Therefore, five AI models were chosen to assess the predictive accuracy of early-age CS in the current study. These models include support vector regression (SVR), random forest (RF), artificial neural network (ANN), gradient boosting (GB), and Gaussian Process Regression (GPR). As part of evaluating model performance and conducting error analysis, this study investigated differences in prediction accuracy among five models across training and testing datasets. Additionally, feature importance analysis was implemented to explore the influence of the input variables on the early-age CS. Results indicate that GPR and SVR models with high predictive accuracy (R^2^ > 0.90) outperformed ANN, RF, and GB models. Water, superplasticizer, curing temperature, and fiber content emerged as the most significant controlling parameters affecting early-age CS. The analysis of the interaction among the significant input variables and early-age CS suggests recommended inclusion levels for optimal performance. Specifically, it is recommended that the water content be maintained between 145 and 155 kg/m^2^, the superplasticizer content between 30 and 40 kg/m^2^, and the fiber content exceed 200 kg/m^2^. These recommendations are aimed at achieving desirable early-age CS characteristics. The overall findings reveal that the AI models can effectively improve the monitoring of early-age CS of UHPFRC.

**Supplementary Information:**

The online version contains supplementary material available at 10.1038/s41598-025-06725-z.

## Introduction

Concrete has remarkable mechanical properties, durability, and cost-effectiveness when compared to other construction materials. Nevertheless, it has certain limitations, including relatively low tensile strength, ductility, and strength-to-weight ratio^1^. To address these limitations, ultra-high-performance fiber-reinforced concrete (UHPFRC) has emerged as a promising solution, significantly improving concrete strength, ductility, and durability. Ultra-high-performance concrete (UHPC), an advanced material based on Portland Cement (PC), is renowned for its exceptional mechanical properties and durability. According to ASTM C1856/C1856M − 17, UHPC is required to achieve compressive strengths (CSs) of at least 100 MPa or 120 MPa. It has become a clear option for strengthening the load-bearing capacity of concrete structures, including bridge decks, prestressed girders, thin walls, and reinforcement layers, in various applications^2,3^. It’s noteworthy that the low water-to-cement (w/c) ratio, typically below 0.2, plays a considerable function in achieving the high packing-density of its constituent particles^3,4^. Consequently, UHPC demonstrates CSs ranging from 60 to 140 MPa within 1 to 7 days. This remarkable early strength facilitates rapid demolding and accelerates construction schedules. However, the complex design principles and unique internal microstructure of UHPC contribute to significant autogenous shrinkage, with a notable portion occurring during the early stages of development. So, various types of fibers have been employed to enhance UHPC’s characteristics, with steel fibers being particularly notable for their ability to enhance fracture properties and CS.

Hence, the existing literature on UHPCs lacks a comprehensive model for predicting the early-age properties, which is considerable for structural design and the overall building process. Construction projects like bridges and skyscrapers, where UHPCs are commonly used, involve complex and lengthy sequences of construction stages. Thus, early-age behavior is an important for long-term performance owing to factors like restraint forces and early-age cracking^5,6^. In other word, early-age CS, a fundamental property of UHPC, guides decisions such as the timing for removing formwork from structural elements and determining the time when concrete pavements can be put into use^7^. To assess the CS of in-situ concrete, it’s common practice to prepare multiple specimens from the mix and subject them to destructive mechanical testing (DMT)^8,9^. However, despite the precision of these CS measurements, they may not accurately reflect the structural strength due to differing curing conditions and stress levels^10^. Furthermore, this DMT approach is difficult and time-intensive, potentially leading to delays in formwork removal and project completion^8^. Hence, there’s a pressing need to develop non-destructive methods (NDM) capable of monitoring early-age concrete strength directly. Recently, a range of NDM have emerged for monitoring the early-age strength evolution of normal concrete^10^and UHPC^11,12^. Normally, during the process of curing concrete, temperature data can be collected using embedded thermocouples or temperature sensors. Subsequently, CS can be estimated using maturity Eqs.^13–15^. For instance, Ibrahim et al.^16^evaluated the accuracy and validity of applying ASTM C1074 procedures for assessing the maturity of UHPC. This evaluation involved testing 5 different UHPC mixtures under various experimental conditions, including different curing regimes, target ages, fiber contents, maturity calculation methods, and specimen shapes and sizes. The assessment revealed that while the ASTM C1074 method could yield satisfactory results some adjustments, and new recommendations were necessary for its effective application to UHPC maturity evaluation. However, this method shows considerable variability in estimated strength when curing happens at different temperatures, even at the same maturity level. Besides that, alternative approaches namely, ultrasonic pulse velocity^12^, rebound hammer testing^17^, and electrical resistivity^18^have found widespread adoption to estimate the CS. These methods depend on measuring specific physical parameters related to concrete strength to establish correlations with strength development. They have demonstrated practical efficiency, user-friendliness, and environmental sustainability in inspections, ensuring structural integrity without causing damage. However, conventional NDM suffers from drawbacks such as being time-consuming, expensive, difficult to operate, and unsuitable for long-term monitoring. Hence, there is an urgent need for prediction models with real-time, autonomous, and intelligent capabilities to accurately monitor and assess the early-age CS of UHPFRC.

## UHPFRC and prediction models: background

Over the last four decades, the development of UHPFRC has motivated structural engineers to improve the CS, ductility, and durability of heavily loaded structures. Researchers have extensively studied the mechanical strength and applications of UHPFRC^19,20^. Typically, UHPFRC demonstrates CSs ranging from 150 MPa to 810 MPa^1,21^. Achieving such high strength requires specific ingredients: a high content of PC, a low w/c ratio, superplasticizer (SP), highly fine cementitious materials like quartz powder (QP), silica fume (SF), and Nano-silica (NS), as well as steel fibers^4,22^. Some researchers have focused on developing sustainable and cost-effective approaches by reducing PC and SF content and substituting them with slag (GGBS)^6^, limestone powder (LP)^23,24^. However, many of these mixtures require significant resources and extensive testing, often resulting in unpredictable UHPFRC strength^25,26^. Thus, there is a critical need for an analytical model that accurately predicts CS based on the UHPFRC constituents.

Various investigations have extensively studied the precise design and forecasting of UHPC and traditional concrete employing artificial intelligence (AI) methods^27,28^. These methods involved meta-heuristic optimization methods like the bald eagle search algorithm and chimp optimization algorithm^29–34^, extreme gradient boosting (XGB)^35,36^, random forest (RF)^35,37^, and Gaussian process regression (GPR) algorithms^36^. For example, Abdellatief et al.^36,38^ developed an XGB model to forecast the CS of UHPC based on cement and geopolymer, achieving prediction accuracies exceeding 0.85. Similarly, a previous study^39^ utilized a machine learning (ML) model with only 110 data points, while Ghafari et al.^25^ developed an ML model based on just 53 samples. However, the small datasets in these studies restrict the generalizability of their models. In addition, Liu et al.^40^ applied support vector regression (SVR) to predict the CS of UHPC using 165 samples, whereas Wu et al.^41^ employed a quadratic regression algorithm based on merely 13 data points. Despite promising results, these algorithms are limited by their ability to handle only a few variables and exhibit low generalization performance due to small datasets, leading to overfitting and reduced prediction accuracy. Furthermore, previous studies have identified significant limitations, including the absence of research on predicting early-age mechanical properties, particularly CS.

## Research objectives and significance

**Fig. 1:**
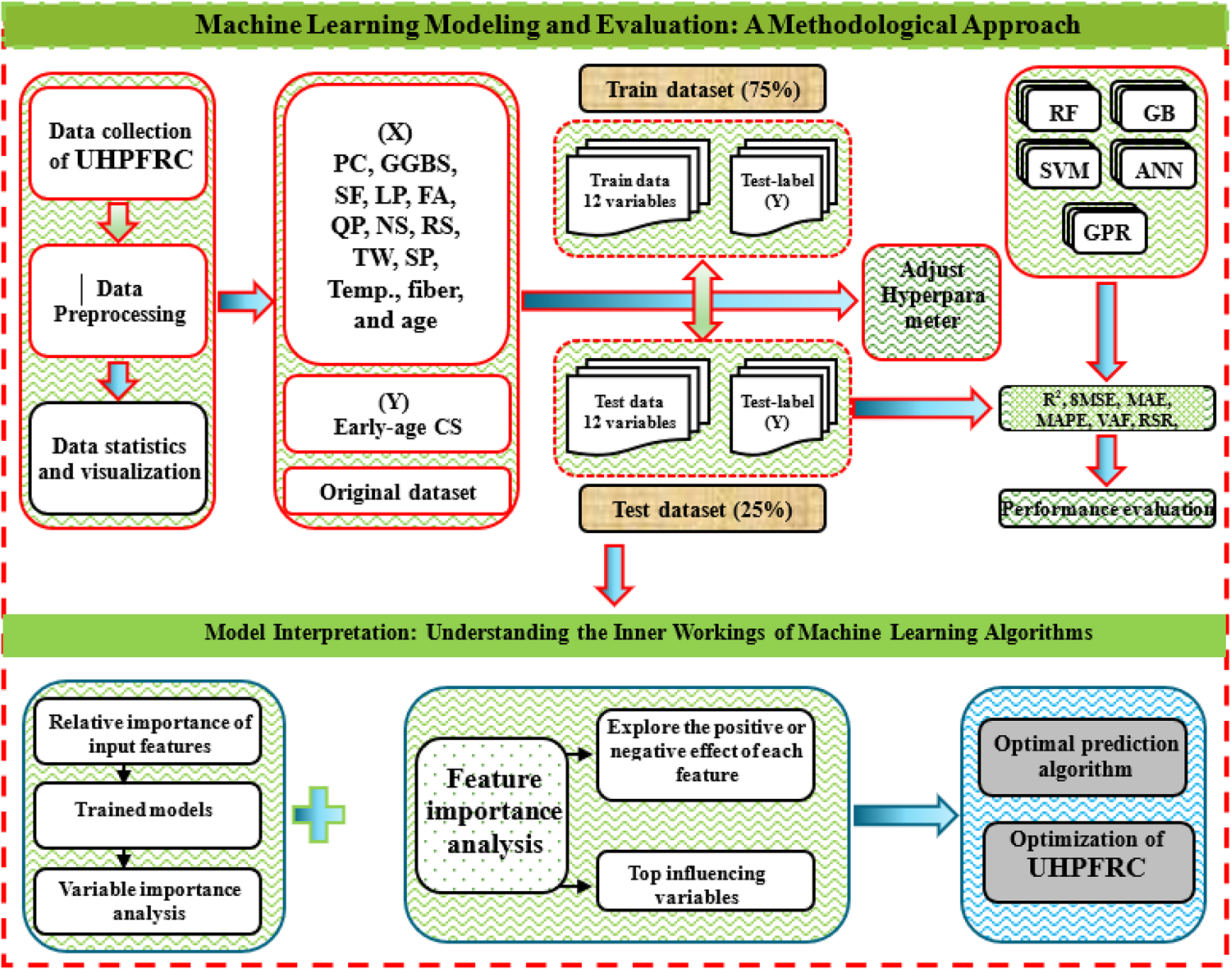
Research methodology overview.

The objective of the current study is to predict the early-age CS of UHPFRC using multiple ML algorithms. 13 input features were considered, including the content of PC, slag (GGBS), silica fume (SF), LP, FA, QP, NS, river sand (RS), tap water (TW), SP, curing temperature (Temp), fiber, and curing age. Five types of ML models, including tree-based models (RF, GB), SVR, GPR, and artificial neural network (ANN) methods were used to forecast the early-age CS of UHPFRC. Finally, feature important analysis was also used to investigate the effect of these variables on CS prediction. By providing insights into early-age CS prediction behavior, this study offers a pathway for their application without extensive experimental work. The general research idea presented in Fig. 1 includes three main parts: training and testing datasets with 5 ML algorithms to determine the optimal model, analyzing the importance of input parameters, and providing initial guidance for mixture design optimization based on the best-performing model.

## Material and method

### Data collection

A comprehensive dataset comprising diverse independent features impacting early-age CS was assembled from the previous literature^4,24,37,40^^42^–^49^. The dataset comprises 293 experimental observations sourced from previously published papers, detailing tests conducted on UHPFRC with various combinations aimed at achieving CS at 1-day, 3-day, and 7-day intervals after casting. Numerous inputs were employed for predicting early-age CS of UHPFRC. Notably, while numerous factors influence early-age CS, only significant ones with sufficient experimental values were incorporated as independent features in this study. The data collection process encountered several challenges, including incomplete and missing data.

### Data statistical summary and parametric study

**Fig. 2:**
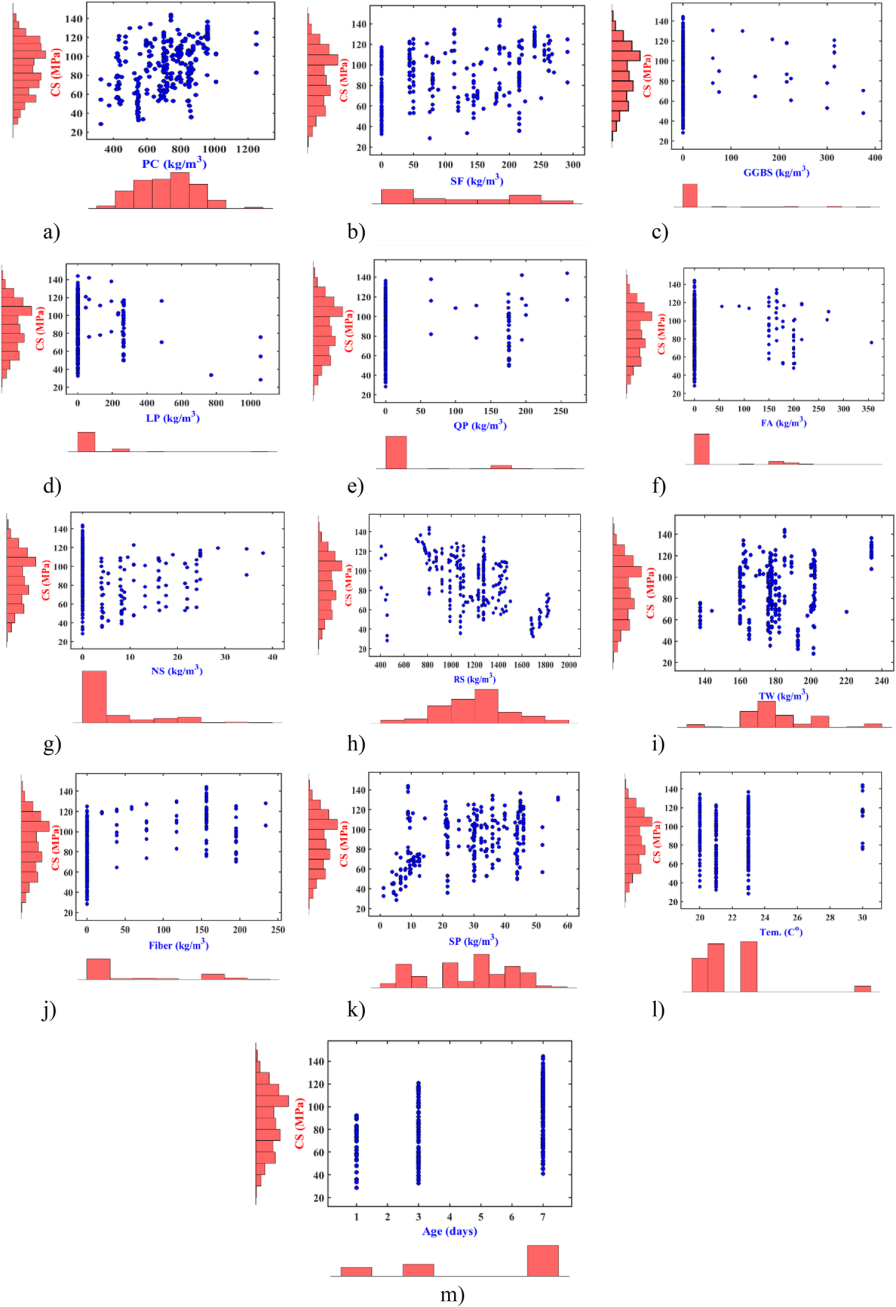
Conjoint distribution plot of independent and dependent variables (**a**) PC, (**b**) SF, (**c**), GGBS, (**d**) LP, (**e**), QP, (**f**), FA, (**g**) NS, (**h**) RS, (**i**), TW, (**j**) Fiber, (**k**) SP, (**l**) temperature, and (**m**) curing age.

Figure 2 indicates the relationship between early-age CS of UHPFRC and 13 input variables. It is observed that the early-age CS ranges from 28.5 MPa to 144 MPa, with values at 1-day ranging from 28.5 MPa to 92.2 MPa, at 3-days from 32.5 MPa to 120.7 MPa, and at 7-days from 40.9 MPa to 144 MPa. Portland Cement (PC, 600–1000 kg/m^2^ for 63% of specimens, Fig. 2a) is the primary binder, driving hydration and forming calcium silicate hydrate (C-S-H), which is critical for early strength development. GGBS (Fig. 2b) shows an inverse correlation with CS, as higher GGBS content (e.g., 60% replacement) reduces early-age CS by up to 50%, consistent with Yalçınkaya et al.^50^, due to its slower pozzolanic reaction. Out of 293 specimens, 118 samples with common SF content ranges between 100 kg/m^2^ to 225 kg/m^2^ (Fig. 2c). Silica Fume (SF, 100–225 kg/m^2^, Fig. 2c) enhances CS by improving particle packing and forming additional C-S-H, though its effect plateaus at higher doses. Most of the LP content (Fig. 2d), approximately 62% of specimens, ranges between 0 and 263 kg/m^2^, while QP (Fig. 2e) and fly ash (Fig. 2f) content range from 150 to 200 kg/m^2^. Fly ash (FA, 150–200 kg/m^2^, Fig. 2f) moderately enhances CS via pozzolanic activity but has a slower reaction than SF. Furthermore, the inclusion of NS does not significantly affect early-age strength (Fig. 2g), whereas RS content plays a considerable parameter in CS (Fig. 2h). The optimal RS dose, around 62% of specimens, ranges between 750 and 850 kg/m^2^. Nearly 73% of samples contain water content within the range of 160–185 kg/m^2^ (Fig. 2i). As expected, there is an inverse correlation between water content and CS. Increased steel fiber content correlates with significant improvements in early-age concrete strength, aligning with previous studies^27,28,41,45,50,51^that indicate the role of fiber in enhancing ductility and bridging cracks (Fig. 2i). It is noted that the inclusion of 156 kg/m^2^ of steel fiber leads to high early-age CS. Additionally, more than 55% of specimens exhibited a SP content ranging between 20 and 40 kg/m^2^ (Fig. 2k). SP content below 20 kg/m^2^ resulted in enhanced early-age CS, reducing setting times and adhering to industry standards. Approximately 88% of samples underwent normal curing at room temperature (Fig. 2l), ranging from 20 °C to 23 °C. Finally, there were 50 samples for one-day curing, 68 samples for three-day curing, and 175 samples cured for seven days (Fig. 2i). Additionally, correlation coefficients for all potential variables have been calculated. They are presented in Table 1, along with statistical data for early-age CS, including Minimum (Min), Maximum (Max), Mean, and Standard Deviation (SD) values^32–34^.

**Fig. 3:**
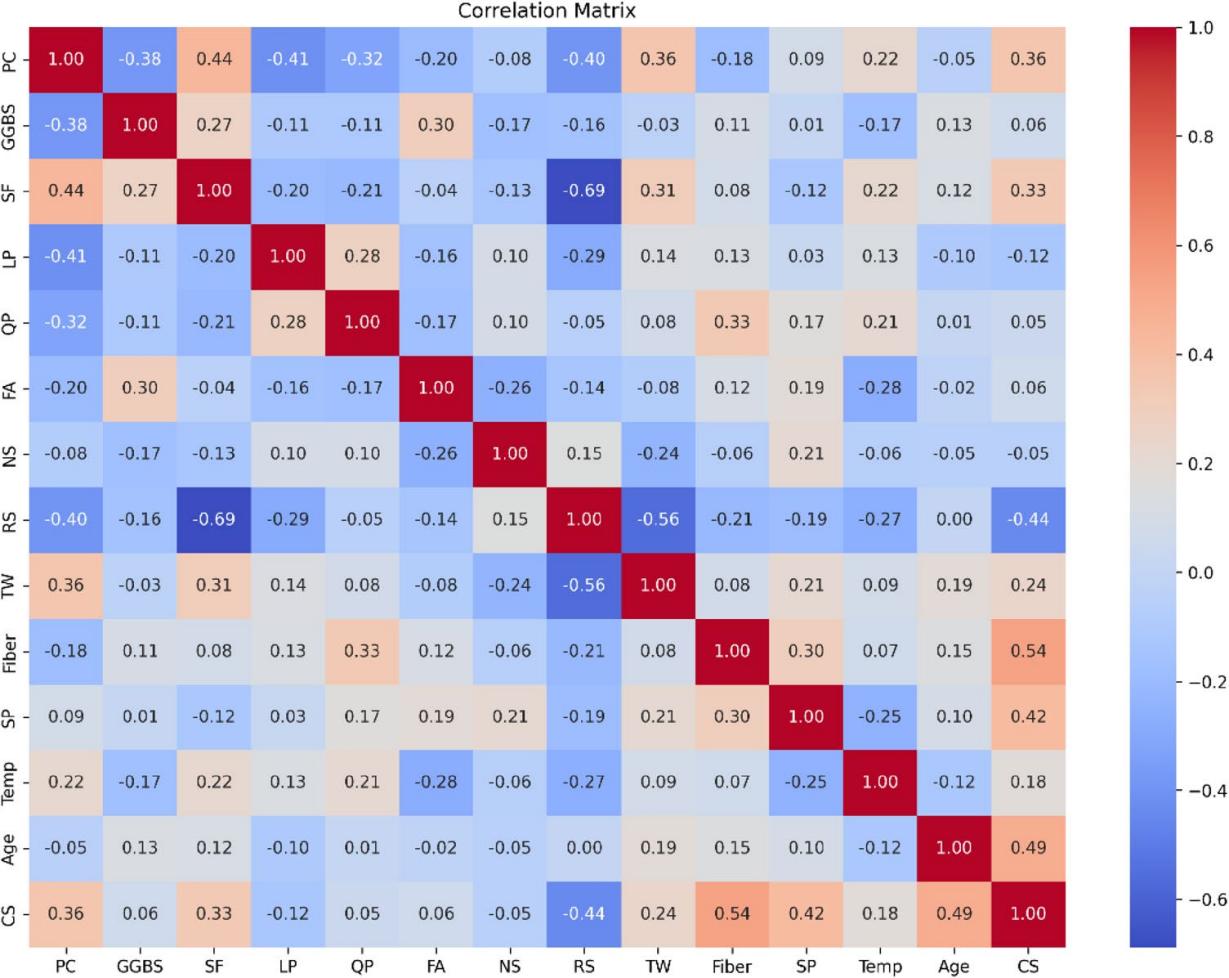
Pearson correlation coefficient matrix.

**Fig. 4:**
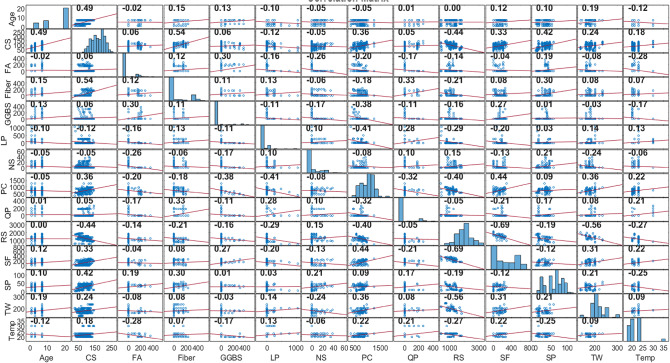
Distribution of the input and output parameters.

Figure 3 exhibits the Pearson relationship coefficients between the input and output parameters, revealing both positive and negative correlations among various factors. Specifically, curing age, SF, TW, and the inclusion of steel fiber demonstrate relatively strong positive relationships with early-age CS, while RS show relatively strong negative correlations. Other influencing factors display comparatively weaker correlations with CS. On the other hand, Fig. 4 illustrates the scatter distribution and the corresponding fitting lines, illustrating the relationship between input and output features. It’s evident that there exists a non-linear correlation among the majority of variables. Additionally, Figs. 3 and 4 indicate that all correlation coefficients between input and output factors are below 0.80, demonstrating the non-linear relations among all input and output parameters. Thus, ML models serve as powerful prediction tools capable of accommodating these nonlinear relationships within the model.

**Table 1:** Characteristics of input and output parameters.

Input and output variables	Unit	Maximum	Minimum	Standard deviation	Mean
PC	Kg/m^3^	1251.2	325.3	170.9	710.2
GGBS		375	0	72.4	20.9
SF		291.3	0	95.7	112.4
LP		1058.2	0	146.7	53.7
QP		259	0	60.2	23.2
FA		356	0	69.8	30.5
NS		38	0	7.96	4.70
RS		1830	407.8	291.1	1184.7
TW		234	137.5	18.8	180
Fiber		234	0	71.1	45.1
SP		57	1.1	13.6	27.8
Temp.	°C	30	20	2.08	21.8
Age	Days	7	1	2.4	5.1
CS	MPa	144	28.5	25.6	87.5

### Constructing AI methods

#### Random forest (RF) method

**Fig. 5:**
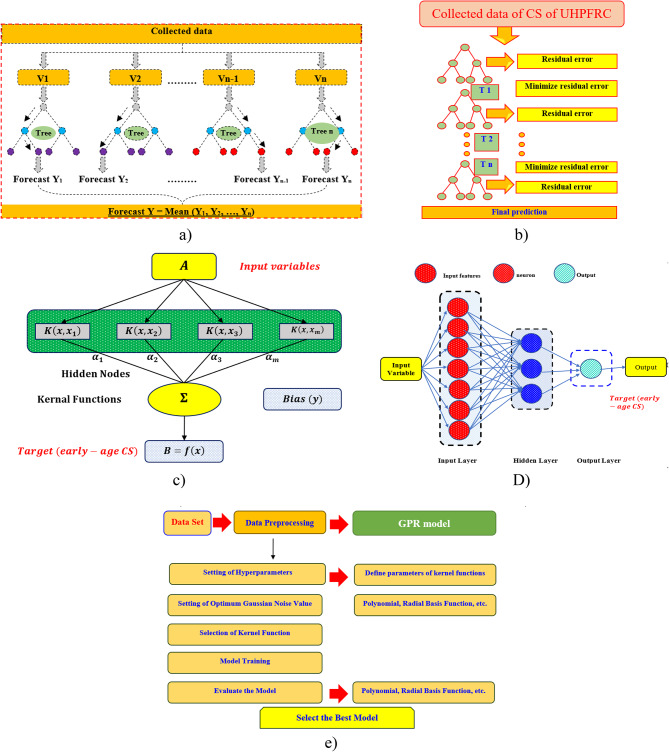
Graphical representation of different ML methods: (**a**) RF, (**b**) GB, (**c**) SVR, (**d**) ANN, and (**e**) GPR.

The RF method is an ensemble learning technique that constructs its predictive framework using multiple decision trees through a bagging approach. This method involves training several decision trees independently on different subsets of the original dataset, and the final prediction is determined by aggregating the outputs of these trees. In RF, a subset of input features is randomly selected to contribute to the formation of each tree, ensuring diversity and reducing overfitting. Originally introduced by^52^, RF falls under the category of bagging-based ML models. It is designed by combining numerous decision trees, each developed employing randomized procedures. As illustrated in Fig. 5a^36,53^, the dataset is partitioned into multiple sub-data points, with each decision tree trained on one of these subsets. During tree construction, the algorithm selects a feature randomly at each node to determine the optimal split into left and right subtrees, which enhances the model’s generalization ability^54,55^. Additionally, the number of trees in the forest plays a crucial role in balancing model accuracy and overfitting. A larger number of trees typically improves the algorithm’s performance by stabilizing predictions and minimizing variance. In RF, predictions are obtained by averaging the outputs of all decision trees, as represented by Eq. (2). Here, Yb refers to the prediction of an individual decision tree trained on the given input X’, while B denotes the total number of trees in the forest.

1 $$\:Y=\frac{1}{B}\sum\:_{j=1}^{b}{Y}_{b}\:\left({X}^{{\prime\:}}\right)$$

#### Gradient boosting (GB) method

The GB method is a ML technique designed for constructing predictive models, particularly ensemble models, by iteratively enhancing weak learners—typically decision trees—through gradient descent optimization. The prediction begins by training an initial base model using the original data points. Afterward, the distribution of the training data is adjusted based on the performance of this base learner, giving higher weight to data points that were previously misclassified^56,57^. This procedure is repeated over multiple iterations, with each new learner refining the mistakes of its predecessors. Eventually, all weak learners are combined to form a strong forecasting method. GB is recognized as a powerful ensemble learning method, widely applied in both classification and regression tasks. Unlike RF, which constructs trees independently using randomization, GB sequentially builds and prunes decision trees in a structured manner to improve accuracy, following the principles of boosting^58^. As depicted in Fig. 5b, the GB algorithm progressively refines predictions by iteratively adjusting its models. The steps involved in its implementation are outlined as follows^36,53^:

2 $$\:{G}_{O}=\sum\:_{i=1}^{N}L({y}_{i},{\upgamma\:})$$

3 $$\:{G}_{m}={G}_{m-1}+argmin\sum\:_{i=1}^{N}\left(L\left({y}_{i},{G}_{m-1}\left({X}_{i}\right)+\:{h}_{m}\left({X}_{i}\right)\right)\right)$$

4 $$\:{\nabla\:}_{\text{L}\text{o}\text{s}\text{s}\:}=-\frac{\partial\:L\left({y}_{i},{G}_{m-1}\left({X}_{i}\right)\right)}{\partial\:{G}_{m-1}\left({X}_{i}\right)}$$

In this context, G_O_ serves as the weak learner, while L represents the loss function. The parameter γ denotes the optimal step size, and y_i_​ corresponds to the actual value of the i-th sample. Furthermore, Gm​ signifies the strong learner, while xi​ stands for the i-th independent feature, and h_m_​ represents the base function. Lastly, ∇Loss refers to the negative gradient of the loss function.

#### Support vector regression (SVR) method

SVR is a ML technique used for regression tasks. It developed by defining a hyperplane in a high-dimensional space that best fits the data points, with the goal of maximizing the margin (distance) between the hyperplane and the closest data points, known as support vectors (Fig. 5c). SVR is effective in capturing nonlinear relationships in data through the use of kernel functions, allowing it to handle complex datasets and achieve accurate predictions^59,60^. SVR excels at delineating classes with distinct margins and proves efficient in memory and space utilization, particularly in high-dimensional datasets. Its primary goal is to identify the hyperplane that optimally segregates samples. The mathematical expression for the hyperplane function is as follows^59^:

6 $$w_{t} x+b=0$$

7 $$\:Minimize\:\frac{1}{2}{‖w‖}^{2}+C\sum\:_{i=1}^{l}\left({\xi\:}_{i}\right)$$

8 $$\:{y}_{i}⟨\varvec{w}.\varPhi\:\:({x}_{a}⟩+b-{y}_{k}\ge\:1-\:{\xi\:}_{ki}^{}\:,\:i=1,\dots\:l$$

9 $$\:{\xi\:}_{i}^{}\ge\:\:,\:i=1,\dots\:l$$.

where, x signifies an n-dimensional vector; w and b are the weight and bias, respectively, ξ_i_ are slack parameters representing the classification error of each sample, C is the penalty parameter controlling the trade-off between maximizing the margin and minimizing the classification error, Φ (x_i_) represents the feature mapping of the dependent vector x_i_, and y_i_ is the class label of the *i*th training instance.

#### Artificial neural networks (ANNs) method

The concept of ANNs originates from early attempts to simulate the sensory processing mechanisms of the human brain^61,62^. An ANN typically consists of three fundamental components: an input layer composed of neurons, one or more hidden layers, and an output layer. These layers are interconnected through weighted connections that influence the flow of information. The ANN operates by first receiving input data through the input layer, which then transmits the information to neurons in the hidden layer. At this stage, no immediate computation occurs. Instead, the neurons in the hidden layer process the incoming signals, extracting relevant patterns and features to establish a functional mapping between the input and output spaces. The processed information is then passed to the output layer, where predictions are generated based on the aggregated knowledge from previous layers^61^. As depicted in Fig. 5d^63^, a standard ANN architecture follows a structured approach. Each neuron first computes a weighted sum of inputs from the preceding layer, with an added bias term. These weights and biases, which determine the strength of connections, are fine-tuned during training. The computed values are then processed using a non-linear activation function, such as the sigmoid function, to introduce non-linearity and improve learning capabilities. During training, the model’s performance is evaluated by minimizing the root mean square error (RMSE) or satisfying predefined convergence criteria.

#### Gaussian process regression (GPR) method

The Gaussian Process Regression (GPR) model is a non-parametric supervised learning technique recognized for its effectiveness in capturing complex nonlinear patterns within datasets. It operates on the assumption that neighboring observations contain valuable information about one another, establishing a prior distribution over the function space. In GPR, a Gaussian distribution (GD) defines the predictive model, where the mean is represented as a vector and the covariance as a matrix. This approach allows GPR to generate a predictive distribution that closely aligns with the test input, enabling it to identify intricate relationships within the data.

Mathematically, GPR consists of a set of random variables that follow a joint multivariate Gaussian distribution for any finite number of observations. Let M and N denote the input and output domains, respectively, with n pairs (M_i_, N_i_) assumed to be independently and identically distributed. In regression tasks, selecting an appropriate kernel function is crucial for defining the structure of the GPR model. Previous studies have explored various kernel functions, and this study adopts the polynomial function within the GPR framework due to its ability to capture nonlinear relationships among input variables^64,65^. Additionally, Fig. 5e illustrates a schematic representation of the GPR method, demonstrating its application in predicting early-age CS^36^.

### Assessing ML models: essential metrics

Table 2 presents the hyperparameters selected for each algorithm, which were fine-tuned using the grid search technique. The dataset was randomly divided into training (75%) and testing (25%) subsets. Hyperparameter optimization was performed using a grid search combined with 5-fold cross-validation on the dataset of 293 UHPFRC specimens to maximize test R^2^ and minimize RMSE. For RF, we tested the number of learners (10–50), minimum leaf size (5–15), and number of predictors sampled, selecting 30 learners, a minimum leaf size of 8, and all predictors to balance complexity and generalization (Table 2). For GB, we varied the number of learners (10–50), minimum leaf size (5–15), and learning rate (0.01–0.5), choosing 30 learners, a minimum leaf size of 8, and a learning rate of 0.1 to ensure stable convergence. SVR optimization involved testing the regularization parameter (C: 100–5000), kernel functions (linear, polynomial, cubic), and kernel coefficient (0.5–2), with C = 1000, cubic kernel, and coefficient = 1 providing robust performance^30,31^. For ANN, we explored activation functions (ReLU, sigmoid), alpha (0.001–0.01), and iteration limits (500–2000), selecting relu, alpha = 0.005, and 1000 iterations to prevent overfitting. GPR was optimized by testing basis functions (constant, linear) and kernel types (isotropic, anisotropic) with automatic signal standard deviation and sigma, retaining the constant basis and isotropic kernel for flexibility in capturing non-linear interactions. Additionally, standard statistical metrics, as detailed in Table 2, were used to comprehensively evaluate the performance of the ML models applied in this study. The performance metrics utilized offer valuable insights into the predictive accuracy of the models, as shown in Table 3.

**Table 2:** Hyperparameter settings of the proposed ML methods.

Model	Parameter	Value
RF	Bagged Tree	–
	Number of Learners	30
	Minimum Leaf Size	8
	Number of Predictors to Sample	Select all
GB	Feature Selection	13/13 individual features selected
	Boosted Tree	–
	Number of Learners	30
	Minimum Leaf Size	8
	Number of Predictors to Sample	Select all
	Learning Rate	0.1
SVR	Regularization Parameter	1000
	Kernel Function	Cubic
	Kernel Coefficient	1
	Feature Selection	13/13 individual features selected
ANN	Activation Function	ReLU
	Alpha	0.005
	Iteration Limit	1000
	Feature Selection	13/13 individual features selected
GPR	Basic Function	Constant
	Use Isotropic Kernel	Yes
	Signal Standard Deviation	Automatic
	Sigma	Automatic

**Table 3:** Performance metrics for evaluating the proposed ML methods.

Definition	Formula
R^2^	$\:1-\frac{{\sum\:}_{i}{\left({y}_{i}-{\widehat{y}}_{i}\right)}^{2}}{{\sum\:}_{i}{\left({y}_{i}-{\stackrel{-}{y}}_{i}\right)}^{2}}$
MSE	$\:\frac{1}{n}\sum\:_{i=1}^{n}{({y}_{i}-{\widehat{y}}_{i})}^{2}$
RMSE	$\:\sqrt{\frac{1}{n}\sum\:_{i=1}^{n}{({y}_{i}-{\widehat{y}}_{i})}^{2}}$
MAE	$\:MAE=\frac{1}{n}\sum\:_{i=1}^{n}\left|{y}_{i}-{\widehat{y}}_{i}\right|$
MAPE	$\:MAE=\frac{1}{n}\sum\:_{i=1}^{n}\left|\frac{{y}_{i}^{{\prime\:}}-{y}_{i}}{{y}_{i}}\right|$

## Results and discussion

### Estimating the performance of ML-Models

**Fig. 6:**
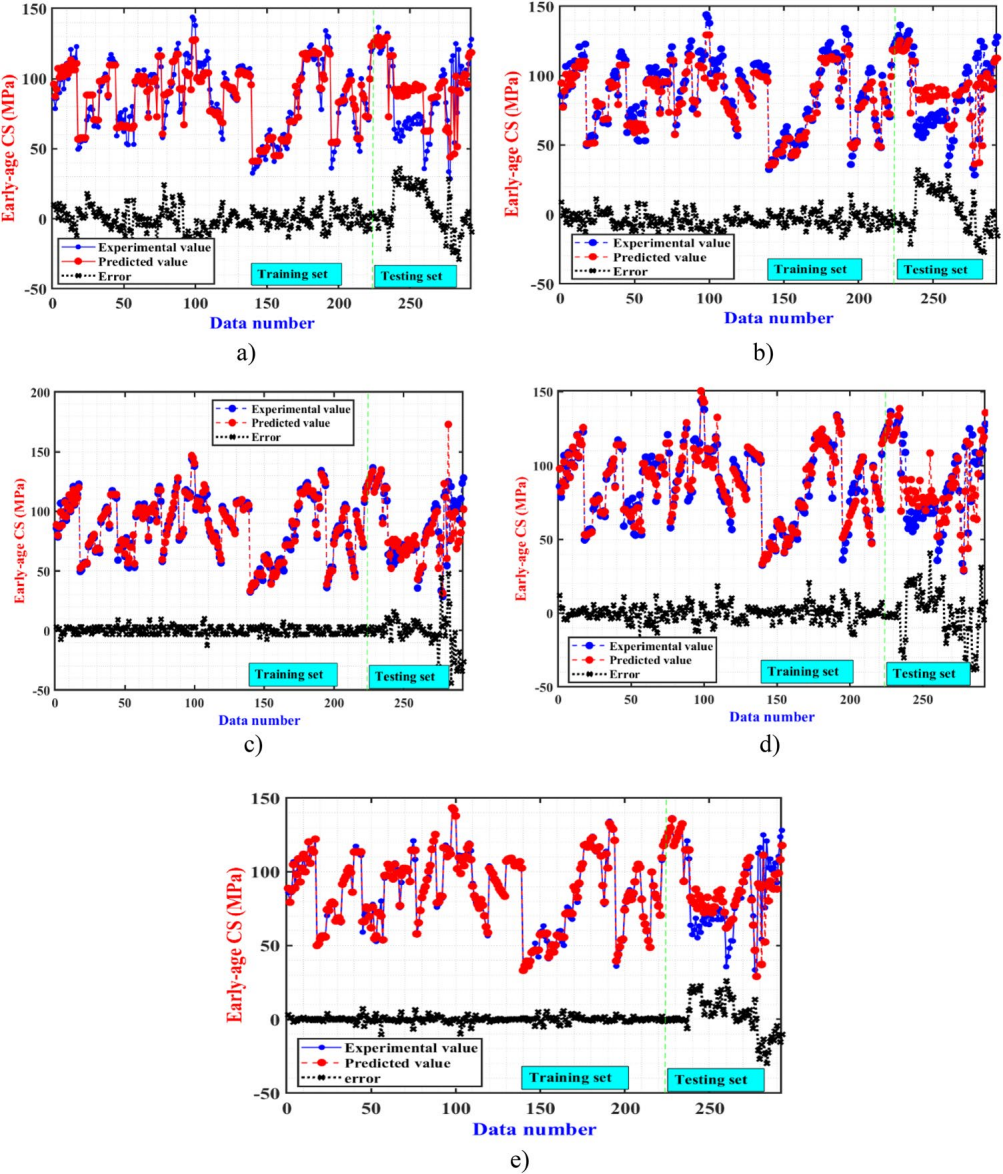
Actual and predicted early-age CS using 5 ML models (**a**) RF, (**b**) GB, (**c**) SVR, (**d**) ANN, and (**e**) GPR model.

Figure 6 shows the predictive results of early-age CS for UHPFRC using the RF, GB, SVR, ANN, and GPR algorithms, respectively. In each figure, the red points represent the actual early-age CS values obtained from experiments, while the blue points denote the predictive early-age CS values calculated by the ML algorithms. Additionally, the black color histograms illustrate the error distribution for each specimen. Observing the training set, it is evident that most samples exhibit relatively small deviations between predictive and actual results, indicating the good performance of the trained algorithms. The MAEs on the train dataset for RF, GB, SVR, ANN, and GPR algorithms are 25 MPa, 15 MPa, 10 MPa, 18 MPa, and 9 MPa, respectively. However, upon examination of the testing set, larger deviations between forecasting and experimental results are noticeable in the figures. The MAEs on the test dataset for RF, GB, SVR, ANN, and GPR models are 36 MPa, 32 MPa, 44 MPa, 43 MPa and 29 MPa, respectively. These findings suggest that all 5 proposed algorithms are capable of effectively predicting the early-age CS for UHPFRC. However, the forecasting performance of the GPR model is confirmed to be relatively better compared to the other algorithms. These results confirm that the primary advantage of GPR lies in its flexibility and ability to provide probabilistic predictions. Unlike many other regression techniques, GPR offers not just point predictions but also estimates of the uncertainty associated with those predictions. This means that GPR not only provides a predicted value for a given input but also quantifies the confidence or uncertainty associated with that prediction^51,65–68^.

### Comparison on prediction performance of five model

#### Statistical analysis of results

**Fig. 7:**
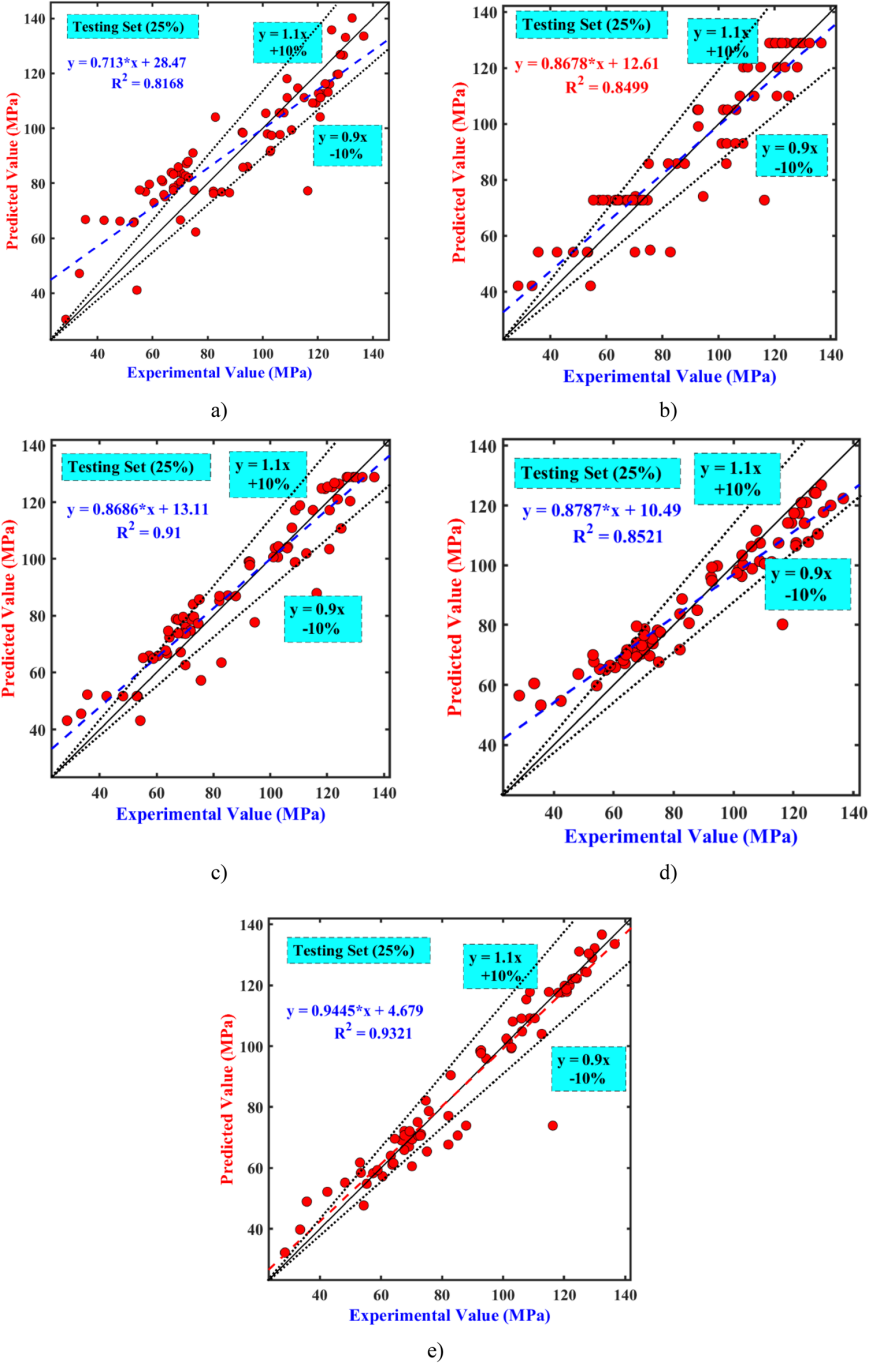
Experimental value vs. predictive value of 5 ML models (Testing dataset) (**a**) RF, (**b**) GB, (**c**) SVR, (**d**) ANN, and (**e**) GPR.

**Fig. 8:**
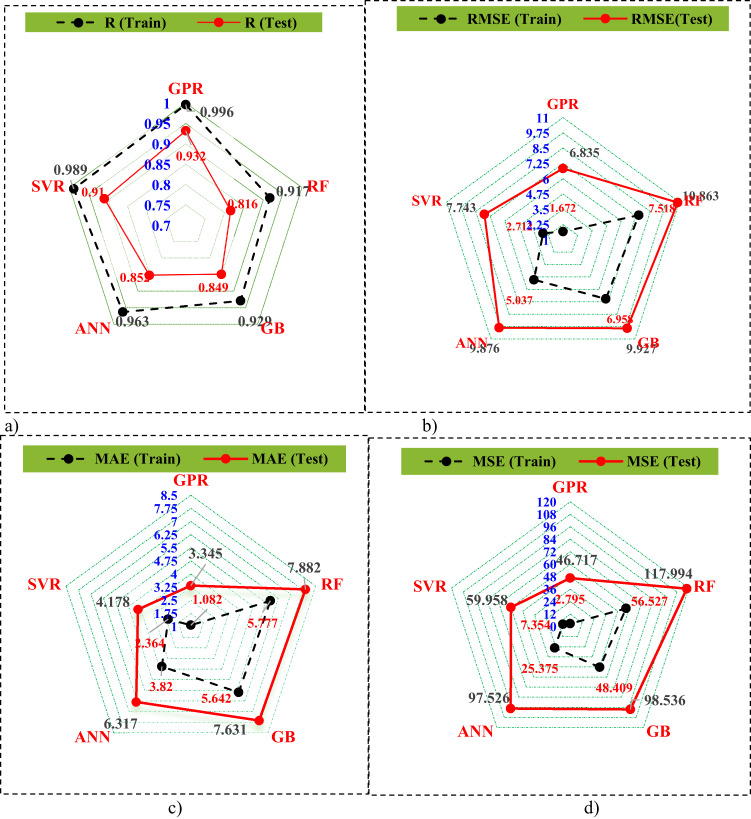
Radar-plot of the evaluation metrics for the 5 algorithms.

The results presented in Fig. S1 and Fig. 7 illustrate notable differences between the performance of various models on both the train and test datasets, respectively. In the training set, except for the tree-based (RF and GB) models, the majority of data points from the other three models closely align along the 45° diagonal, yielding a remarkably high correlation coefficient of 95–99% between experimental and predicted values. However, on the testing set, apart from RF, data points from another algorithms cluster closely around the centerline, predominantly falling within a 10% error margin. Particularly among the proposed models, GPR, SVM, and ANN, there are notably fewer testing data points exceeding the ± 10% error thresholds. These findings demonstrate the GPR, SVM, and ANN models’ superior generalization capability compared to tree-based models in this analysis. Table 4 presents the performance evaluation metrics for 5 models. A higher R^2^ indicates better alignment of the algorithms with the datasets, demonstrating strong generalization ability. On the contrary, smaller results for MAE&RMSE suggest better algorithm accuracy. In Table 4, the GPR model demonstrates the strongest generalization ability in the training dataset, with consistent high R^2^ values (0.99) and low RMSE, MSE, and MAE values (1.67, 2.79, and 1.08, respectively). The SVM and ANN models also exhibited impressive training performance compared to the RF and GB tree-based models. In the test set, the GPR model achieved the highest R^2^ (93.2%) and the lowest RMSE, MSE, and MAE values (6.83, 46.71, and 3.34, respectively). The following best performing algorithms are the SVR and ANN algorithms, which have R2 values of 91% and 85%, respectively. However, the tree-based models display poor prediction accuracy on the test phase. Of all the models, the RF model shows the worst generalization ability and prediction accuracy. To further evaluate the performance of the 5 algorithms, Fig. 8 illustrates the evaluation indexes for the 5 ML-models on both train and test phases. The GPR approach permanently shows higher R^2^ values (Fig. 8a) and lower RMSE, MSE, and MAE values (Fig. 8b) during both phases. These results validate the model’s reliability and robustness, confirming its accuracy and ability to generalize well. It was also found that the GPR approach surpasses RF, GB, ANN, and SVR approaches, demonstrating improvements of 12.44%, 8.9%, 8.58%, and 2.36% in R^2^, and reductions of 4.54%, 4.29%, 2.97%, and 0.83% in MAE, and 4.02%, 3.09%, 3.04%, and 0.91% in RMSE, respectively (Fig. 8c and d).

**Table 4:** Performance metrics summary for training and testing datasets across models.

Model type	Status	RMSE	MSE	*R* ^squared^	MAE
RF	Trained	7.518	56.527	0.917	5.777
	Tested	10.863	117.994	0.816	7.882
GB	Trained	6.958	48.409	0.929	5.642
	Tested	9.927	98.536	0.849	7.631
SVR	Trained	2.712	7.354	0.989	2.364
	Tested	7.743	59.958	0.910	4.178
ANN	Trained	5.037	25.375	0.963	3.820
	Tested	9.876	97.526	0.852	6.317
GPR	Trained	1.672	2.795	0.996	1.082
	Tested	6.835	46.717	0.932	3.345

#### Regression error characteristic (REC) curve

**Fig. 9:**
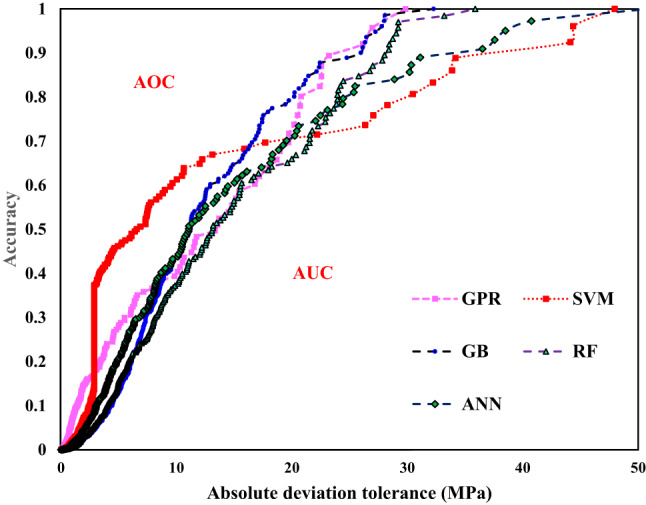
REC curve for different ML models.

Receiver Operating Characteristic (ROC) curves are graphical tools used to efficiently compare and display classification values. Recently, they have been adapted for regression analysis into REC curves. Figure 9 illustrates the REC plot for the five ML models, highlighting the superiority of the GPR model with the lowest area over the curve (AOC) value. Conversely, the RF and ANN models achieve the highest AOC values among all models. REC plots depict the percentage of accurately forecasted instances within a tolerance interval against the absolute deviation tolerance. They offer insights into the cumulative distribution function of errors. The AOC provides an estimate of predicted error, derived from the area under the curve (AUC). preferably, a perfect regression model would have a low AOC value, with the curve aligned parallel to the y-axis, as achieved by the GPR model^30,34^.

**Fig. 10:**
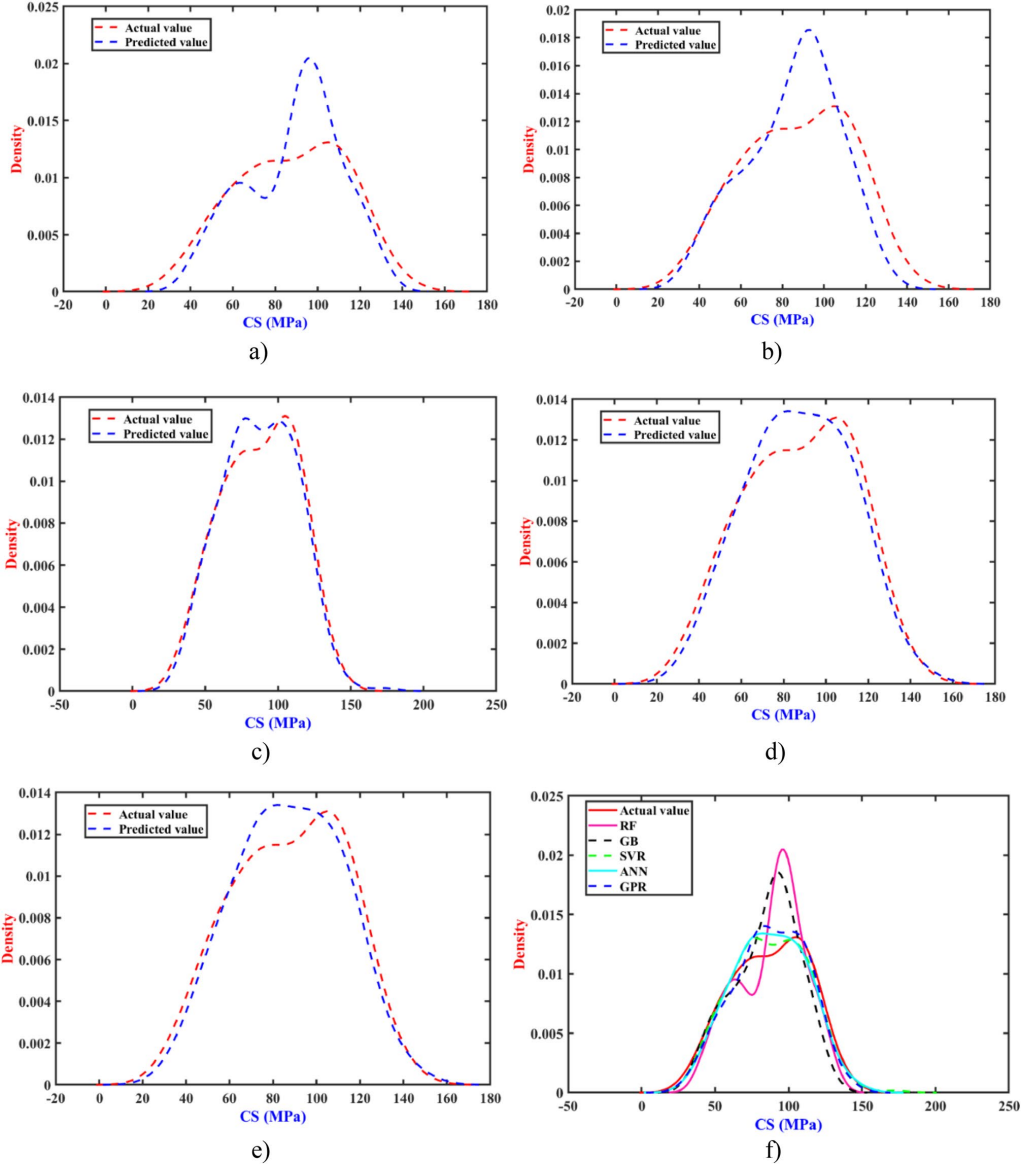
Density distribution plot of experimental and predicted values (**a**) RF, (**b**) GB, (**c**) SVR, (**d**) ANN, (**e**) GPR, and (**f**) 5 ML models.

The error distribution of actual early-age CS values and forecasted CS values of UHPFRC of all the algorithms were plotted, as shown in Fig. 10. It was observed that the errors from these ML models exhibit a normal distribution. Except for the RF and GB models (Fig. 10a and b), the error distribution for the other algorithms was minimal, especially in the GPR model (Fig. 10e). In particular, the GPR model demonstrates the ability to predict the early-age CS of UHPFRC with high confidence within a narrow range. Thus, the GPR model characterizes the highest prediction accuracy and delivers more resilient results even amidst lower levels of uncertainty (Fig. 10f).

#### Effect of independent features sensitivities

**Fig. 11:**
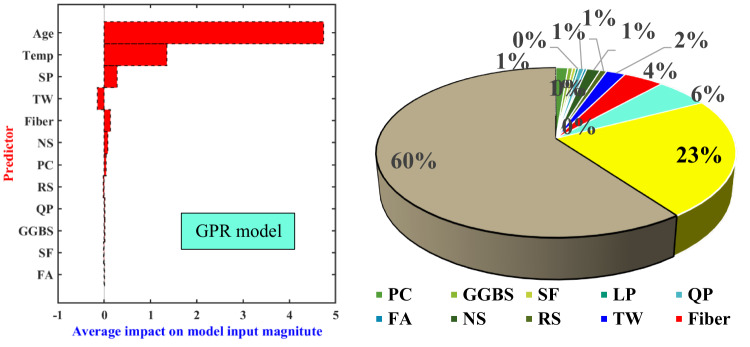
Feature importance analysis based on the GPR model.

Based on the findings outlined in Section “Statistical analysis of results”, the best model demonstrating the most exceptional predictive performance for early-age CS was selected for a relative importance analysis of input features. Feature importance analysis serves as a valuable tool for understanding the extent to which each independent feature contributes to the dependent variable. Given the superior predictive performance of the GPR model, this study examines the feature importance of independent variables based on the GPR algorithm, as illustrated in Fig. 11. The age parameter emerges as the most influential factor affecting the early-age CS of UHPFRC, attributing to 60.6% of the variance. It makes some sense since we can observe from Fig. 2 that there is a dramatic growth of the CS during 1 day to around 7-d. Additionally, temperature curing, SP, fiber content, and water content also wield considerable influence, accounting for 23.1%, 6.06%, 4.1%, and 2.1% respectively. In contrast, variables such as PC, RS, QP, GGBS, and FA exhibit minimal impact on early-age CS, each contributing less than 1%. Consequently, adhering to Occam’s razor principle, which reported for simplicity, it may be wise to consider reducing the inclusion of these less impactful variables in future studies. This strategic reduction can streamline the ML model’s complexity, thereby mitigating the risk of overfitting and enhancing its robustness.

#### Partial dependence plot analysis (PDP)

**Fig. 12:**
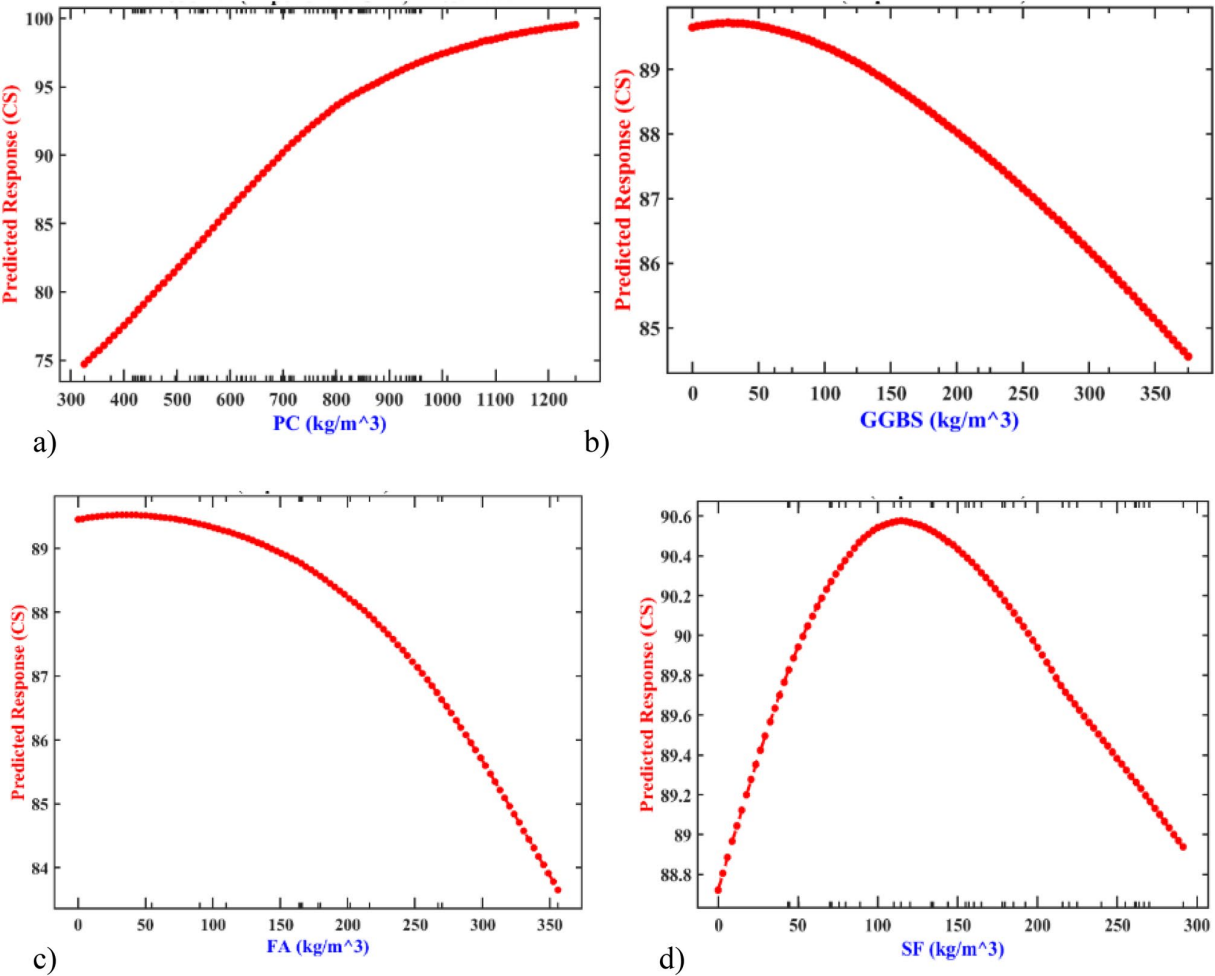
PDP analysis of the effects of independent variables on CS (**a**) PC, (**b**) GGBS, (**c**) FA, and (**d**) SF.

To better understand the factors influencing the early-age of CS at UHPFRC, a method called the partial dependence plot (PDP) was employed. As presented in Fig. 12 and Fig. S2, the analysis revealed that the content of supplementary cementitious materials (SCMs) has a negative impact on the early age of CS, whereas PC, age, curing temperature, NS, and curing age have a positive relationship with the early age of CS. Lowering the water content, optimum SP dose, and increasing curing age up to 7-d significantly enhance the early age of CS. Specifically, PC content ranging from 300 kg/m^3^ to 1200 kg/m^3^ can achieve early-age CS values of 75 to 100 MPa (Fig. 12a). Conversely, increasing GGBS (Fig. 12b) and FA (Fig. 12c) content from 0 kg/m^3^ to 350 kg/m^3^, decreases early-age CS. Increasing SF content up to 120 kg/m^3^ enhances the early-age CS due to the presence of tiny SF particles (Fig. 12d)^69^. Xi et al.^70^found that the inclusion of SF facilitates the acceleration of PC hydration primarily due to additional nucleation sites for the hydration products, known as the seeding effect. Additionally, increasing NS content up to 16 kg/m^3^ (Fig. S2a) effectively enhancing the packing-density of UHPFRC, filling voids between micro-scale components^71^. However, excessive use of RS, LP, and QP content leads to a decline in the early age of CS (see Fig. S2b to d). Additionally, the optimum content of the SP ranges from 30 kg/m^3^ to 40 kg/m^3^ as presented in Fig. S2e. Furthermore, increasing fiber content enhances the early age CS, as demonstrated in recent studies (Fig. S2f), wherein steel fibers act as reinforcements bridging cracks in the matrix under loading, reducing crack propagation and controlling microcrack formation within the concrete matrix^24,41^. Finally, as presented in Fig. S2g, the early-age CS exhibited an increase as the w/c ratio decreased. This trend aligns with Abrams’ law observed in PC-based composites, which suggests that strength increases as the w/c ratio decreases. Previous studies have demonstrated that during the initial stages of curing, the hydration of cementitious materials, including PC and SCMs, leads to the formation of C-S-H gel and other hydration products. This early phase has an important function in the initial strength development, often resulting in noticeable strength improvements within the first few days of curing. As curing progresses, ongoing hydration reactions continue to refine the microstructure of UHPFRC. This process leads to increased density, decreased porosity, and enhanced interfacial bonding between PC particles and aggregates (Figs. S2h and i). Consequently, this densification process contributes to the continuous enhancement of UHPFRC’s early-age strength over time. Typically, UHPFRC achieves significant strength development within the initial 7 days of curing which reaches above 100 MPa, with additional strength gains occurring beyond this period.

#### Experimental validation and comparative analysis with previous studies

**Fig. 13:**
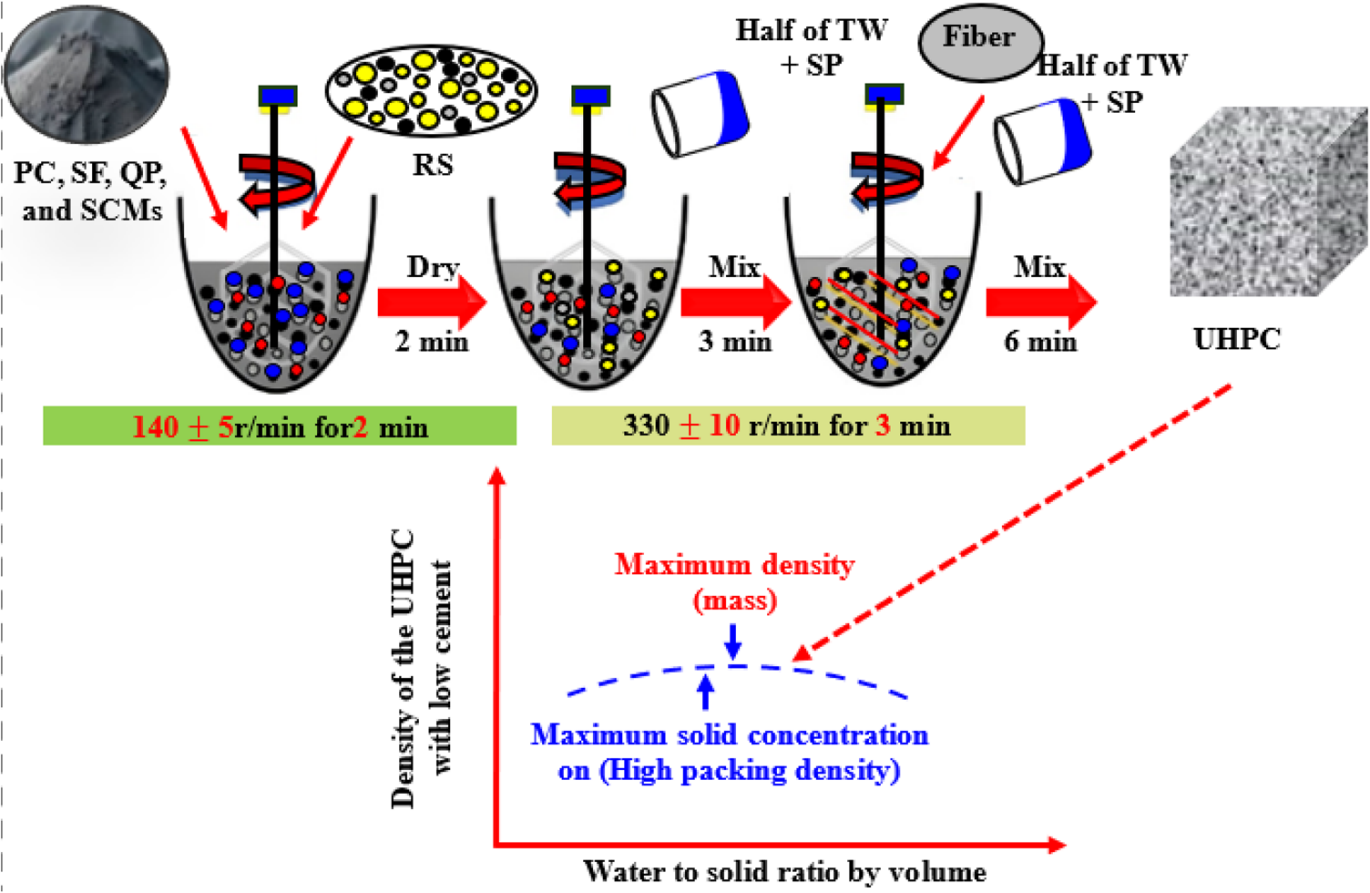
Schematic diagram of UHPFRC synthesis process.

To validate the accuracy of the findings obtained through ML approaches in this study, UHPFRC mixtures with steel fiber were prepared in previous research^4,24^. The effects of various factors, including GGBS, FA, fiber content, and curing age, on the CS of UHPFRC were thoroughly investigated. Some early-age CS test results were referenced from previously published work, and the detailed synthesis procedure is outlined in Fig. 13, with the early-age CS results presented in Table 5. The experimental findings reveal that the increasing of GGBS content decreases the early-age CS as well as the strength enhanced with the curing time. Additionally, a higher FA content exhibits a significant adverse impact on the early-age CS of UHPFRC. Conversely, curing age exhibits significant effect on the early-age CS of UHPFRC. These experimental results align well with the outcomes obtained from the current results of ML-models, confirming the applicability of such methods in the performance analysis of UHPFRC. Additionally, the current study on UHPFRC demonstrates superior predictive performance compared to other studies on CS prediction. Using five ML models, our GPR model achieved the highest R^2^ of 0.932 and the lowest RMSE (6.835) and MAE (3.345), outperforming Reference^72^’s best model (ANN + SVR + LR, R^2^=0.883, RMSE = 8.36) for normal concrete and Reference^35^’s best model (RF, R^2^=0.85, RMSE = 9.335) for UHPC. Compared to Reference^73^, which reported a higher R^2^ (0.978) with DMO-CatBoost for UHPC, our study’s GPR and SVR (R^2^=0.91) models still show competitive accuracy with simpler models. Overall, our models, particularly GPR, provide robust predictions for early-age CS in UHPFRC, achieving lower errors than most models in the referenced studies.

To benchmark the performance of our ML models, we compared the early-age CS predictions of UHPFRC against traditional empirical models, including Abrams’ law and equations from the American Concrete Institute (ACI). Abrams’ law relates CS to the water-to-cement (w/c) ratio, while ACI models estimate CS based on curing age and mix proportions. When applied to our dataset, Abrams’ law and the ACI equations achieved R^2^ values of 0.65 and 0.72, respectively, with corresponding RMSE values of 15.2 MPa and 13.8 MPa. In contrast, our GPR model yielded a significantly higher R^2^ of 0.932 and a lower RMSE of 6.835 MPa, while the SVR model achieved an R^2^ of 0.910 and RMSE of 7.743 MPa (as detailed in Table 6). These results demonstrate the superior predictive capability of ML models in capturing nonlinear interactions among 13 input variables, including fiber content and curing temperature, which are not effectively represented in traditional empirical formulations.

**Table 5:** Early-age CS results for the UHPFRC samples^4^.

Mix ID	PC	SF	SCMs	QP	RS	SP	Fiber	TW	CS (MPa)	
	(Kg/m^3^)								3-d	7-d
1	900	135	–	180	1271	24	156	166	80	85
2	630		270						70	80
3	540		360						63	77
4	450		450						55	63
5	720		180						78	83
6	675		225						77	81
7	630		270						64	79
8	765		135						85	89
9	720		180						79	88
10	675		225						64	74

**Table 6:** Performance evaluation of different ML models in CS prediction (testing phase).

Ref.	Concrete type	(ML) models	RMSE	MAE	*R* ^2^
Current study	UHPFRC	RF	10.863	7.882	0.816
		GB	9.927	7.631	0.849
		SVR	7.743	4.178	0.91
		ANN	9.876	6.317	0.852
		GPR	6.835	3.345	0.932
^72^	Normal concrete at early age	ANN	7.176	5.55	0.909
		SVR	10.91	8.687	0.779
		LR	10.97	8.634	0.768
		ANN + SVR + LR	9.039	7.103	0.855
		ANN + SVR + LR	8.36	6.622	0.883
		ANN + LR	8.319	6.504	0.876
^73^	UHPC	CatBoost	6.4	4.88	0.96
		PPSO-CatBoost	6.23	0.039	0.976
		DMO-CatBoost	6.15	0.038	0.978
^35^	UHPC	MLP-ANN	11.734	8.187	0.77
		DT	11.014	7.285	0.8
		RF	9.335	6.485	0.85
		SVR	9.67	6.971	0.84
		KNN	10.63	7.67	0.81
		BR	15.308	12.294	0.61

Linear regression (LR), Particle Swarm Optimization-CatBoost (PPSO-CatBoost), and Differential Mutation Optimization-CatBoost (DMO-CatBoost), Multi-Layer Perceptron-ANN (MLP-ANN), Decision Tree (DT), RF, SVR, K-Nearest Neighbors (KNN), and Bayesian Ridge (BR).

## Conclusions

This study has applied five advanced machine-learning techniques to predict and optimize the early-age strength of UHPFRC, leveraging a comprehensive dataset with 13 diverse input parameters. The findings reveal several key insights: Critical factors such as SF content, fiber, PC content, SP dosage, RS presence, curing temperature, and concrete age have a strong positive correlation with early-age CS, with higher values generally enhancing strength. Conversely, RS shows a negative correlation, while factors like GGBS, FA, NS, QP, and LP dosage have a relatively minor impact. A detailed analysis demonstrates the significance of SF and SP in achieving high early-age CS, with optimal ranges identified between 80 and 120 kg/m^2^ for SF and 25–45 kg/m^2^ for SP. Among the machine learning models evaluated, SVR and GPR demonstrated outstanding predictive accuracy, both achieving R^2^ values above 0.90 for training and testing datasets. Notably, GPR emerged as the top-performing model, exhibiting the highest R-values and the lowest RMSE, MSE, and MAE metrics, thereby establishing its superiority in early-age CS prediction. While all models showed strong predictive capabilities with scatter plots closely aligned with the ideal diagonal line, the RF model exhibited slightly lower accuracy. Nevertheless, the majority of predictions across all models remained within an acceptable error margin of ± 10%, confirming the robustness and reliability of the proposed modeling approach.

## Limitations and future work

Despite strong model performance—particularly from GPR and SVR—several limitations remain. The dataset originates from limited lab-scale experiments and existing literature sources which prevents drawing broad conclusions. The model’s robustness would benefit from using large-scale datasets that cover diverse mix designs and curing conditions. The identification of major early-age CS variables occurred but the model did not include potential interactions between SP and SF which require analysis through response surface methodology. The analysis did not account for the long-term effects related to shrinkage and durability and sustainability factors. To improve the interpretability of models it is important to include explainable AI techniques such as SHAP or LIME. The field application of models requires real-world validation to establish their effectiveness. The reliability and practical utility of machine learning in UHPFRC mix optimization will be enhanced by addressing these gaps.

## Electronic supplementary material

Below is the link to the electronic supplementary material.

Supplementary Material 1

## Data Availability

All data generated or analyzed during this study are included in this published article as supplementary information files. In addition, any additional required data is available by the corresponding author. The corresponding author is Hassan Hamouda from the Faculty of Technology and Education, Suez University. The research funding was provided through the Egyptian Knowledge Bank (EKB), and confirmation of this support can be obtained via the official email: Hassan.hamouda@ind.suezuni.edu.eg.
