# Supplementary material for: AI driven prediction of early age compressive strength in ultra high performance fiber reinforced concrete
Source: Sci Rep. 2025 Jun 26;15:20316. doi: 10.1038/s41598-025-06725-z (PMC12202810; doi:10.1038/s41598-025-06725-z)
Supplement: Supplementary file 1 — Supplementary Material 1 [file 41598_2025_6725_MOESM1_ESM.docx]

| 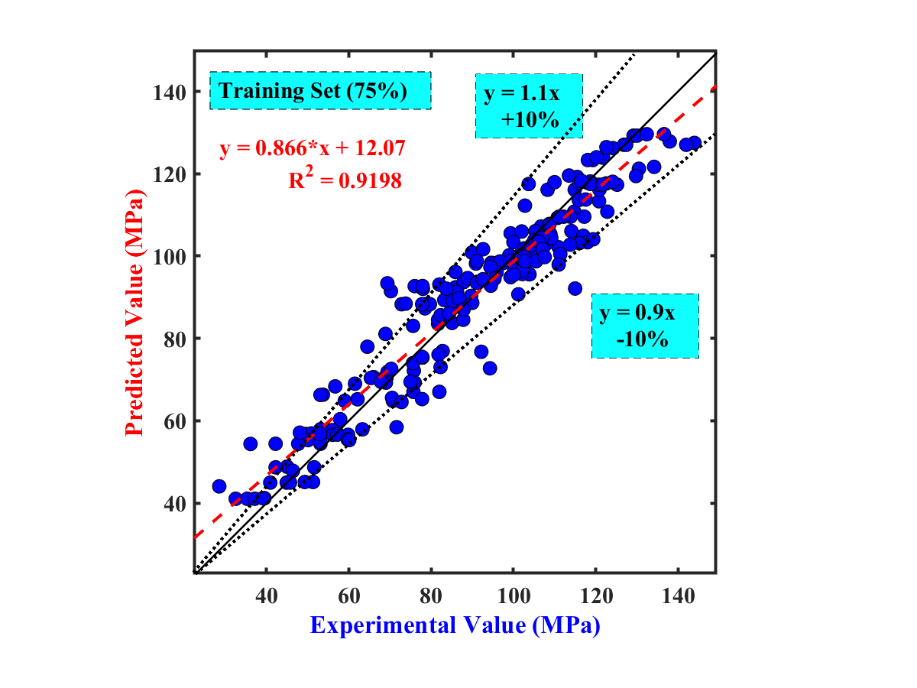 | 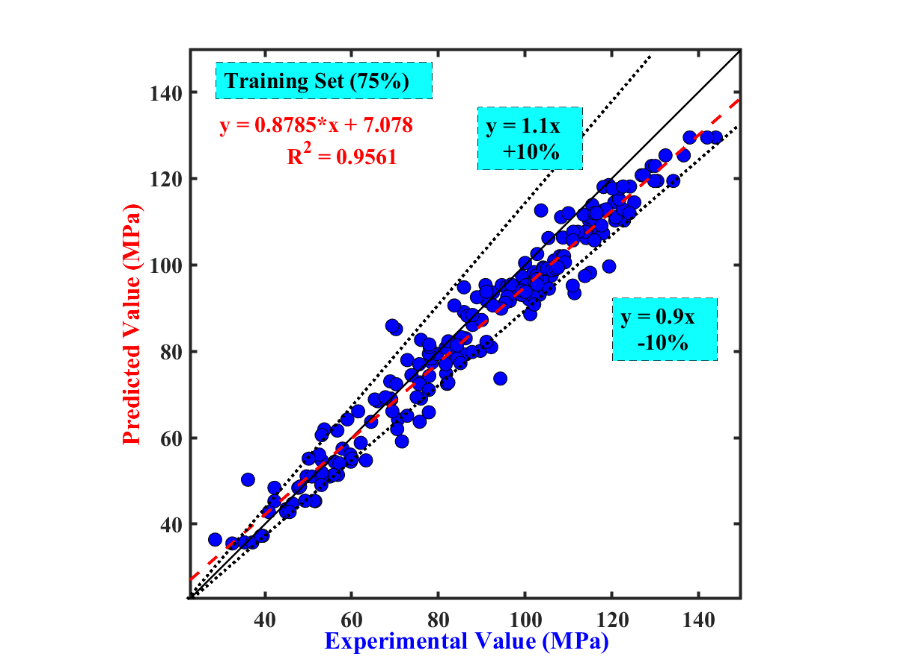 |
| --- | --- |
| 1. RF (Training set) | 1. GB (Train dataset) |
| 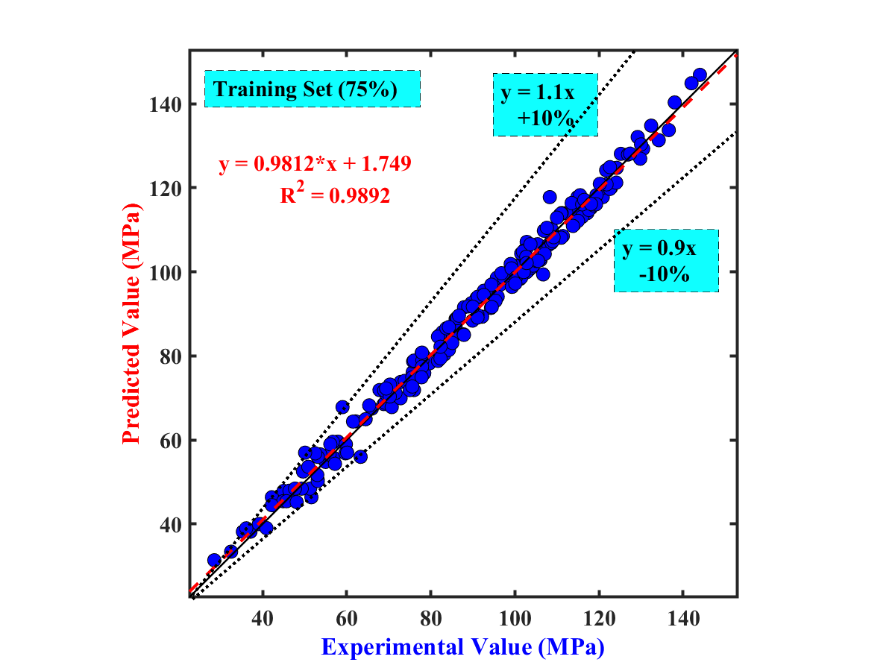 | 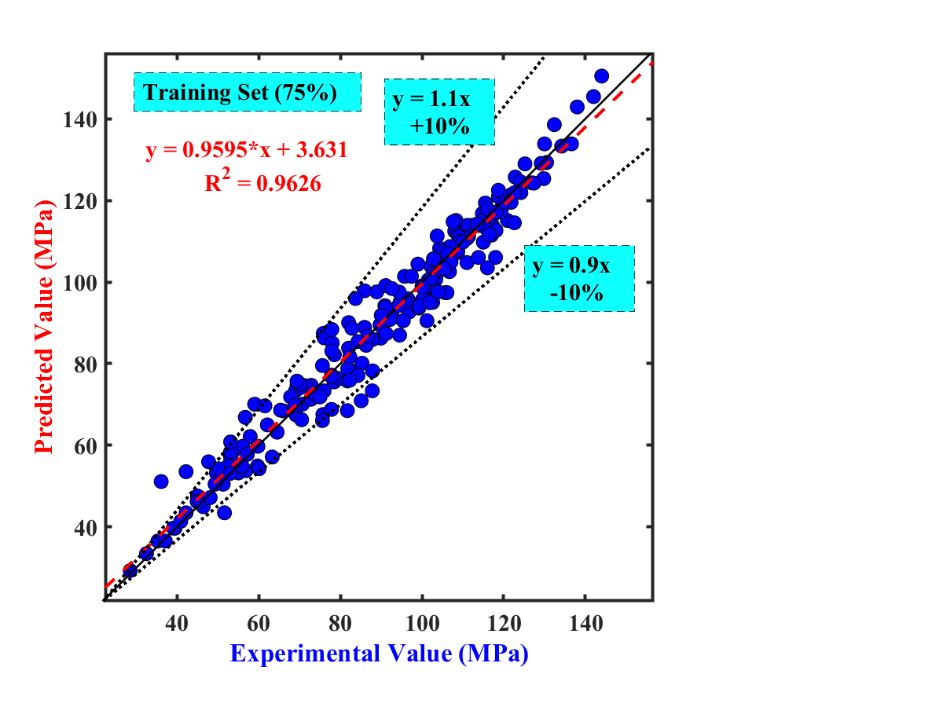 |
| 1. SVR (Train dataset) | 1. ANN (Train dataset) |
| 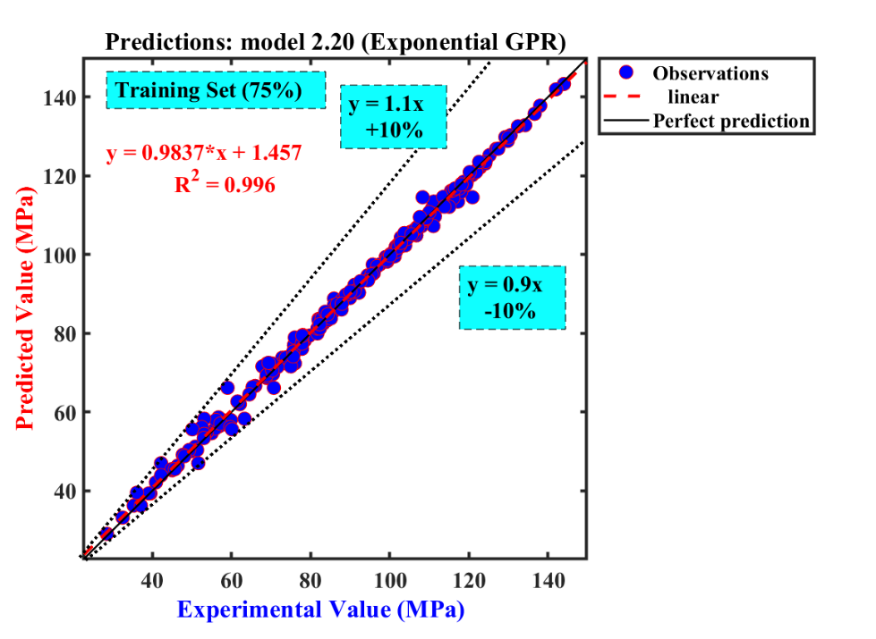 | |
| 1. GPR (Train dataset) | |

Fig. S1**.** Experimental value vs. predictive value of 5 ML models (Training dataset).


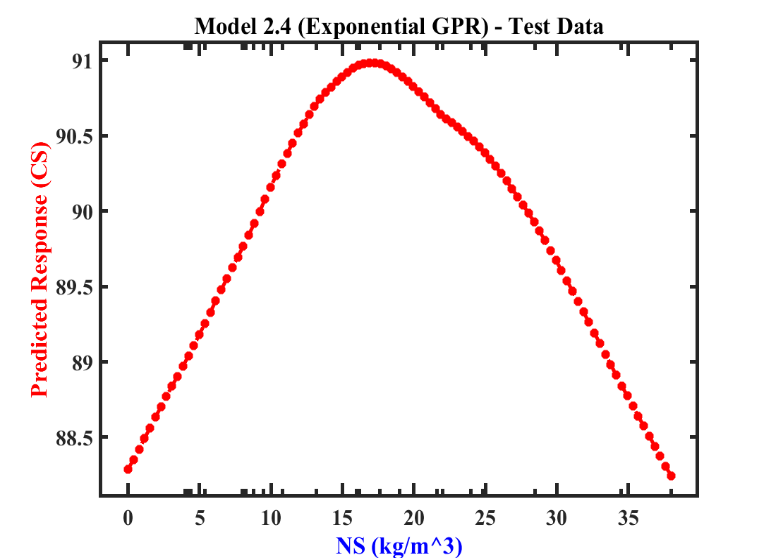

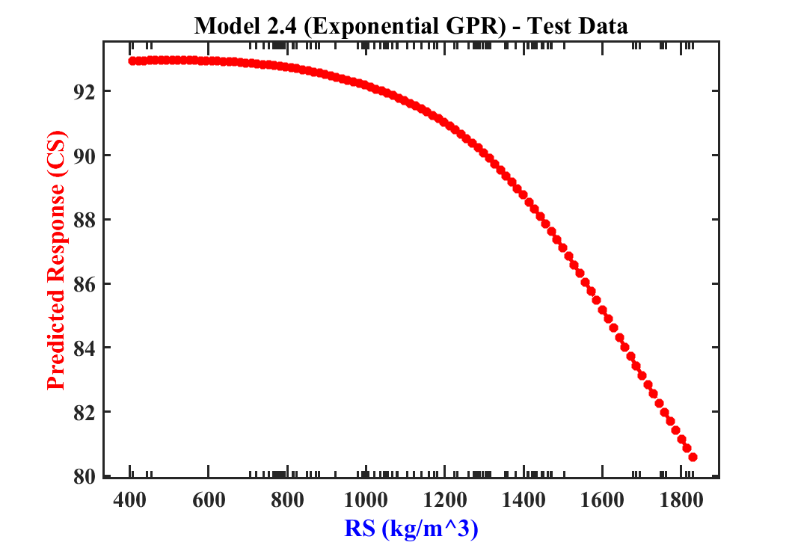


1. b)


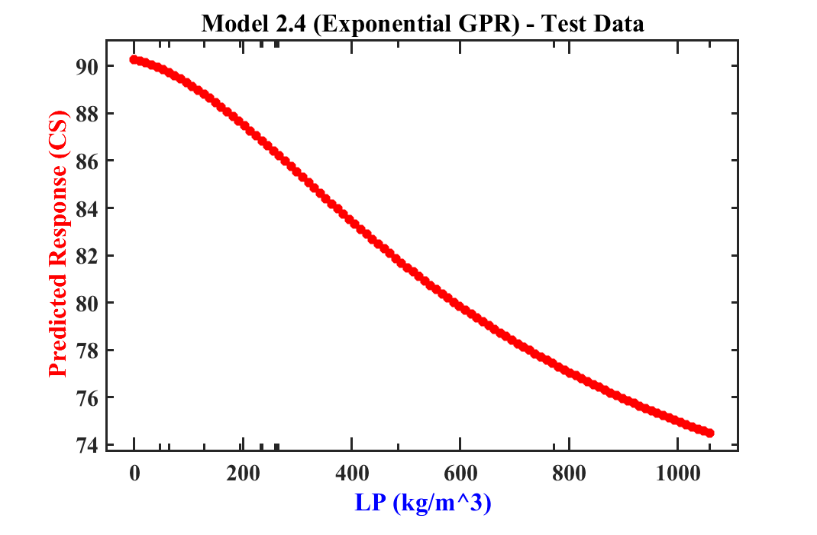

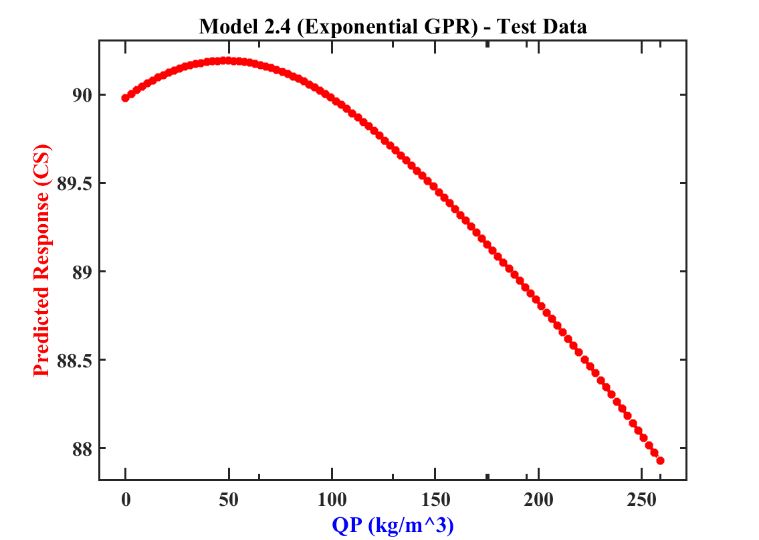


c) d)


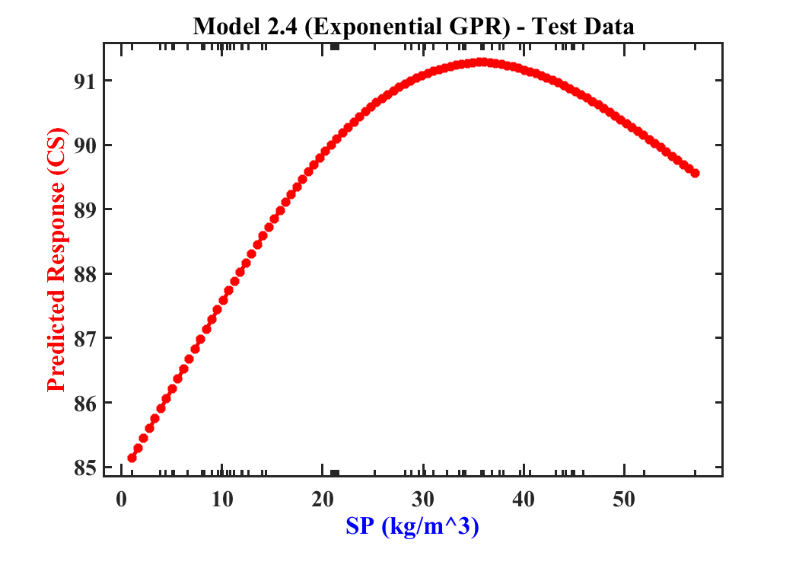

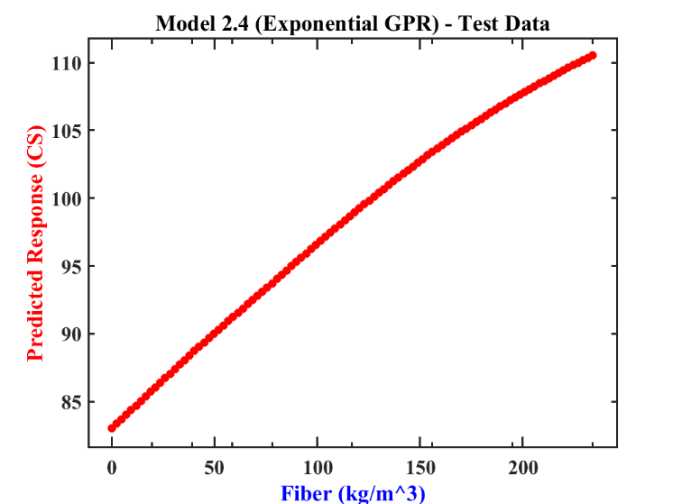


e) f)


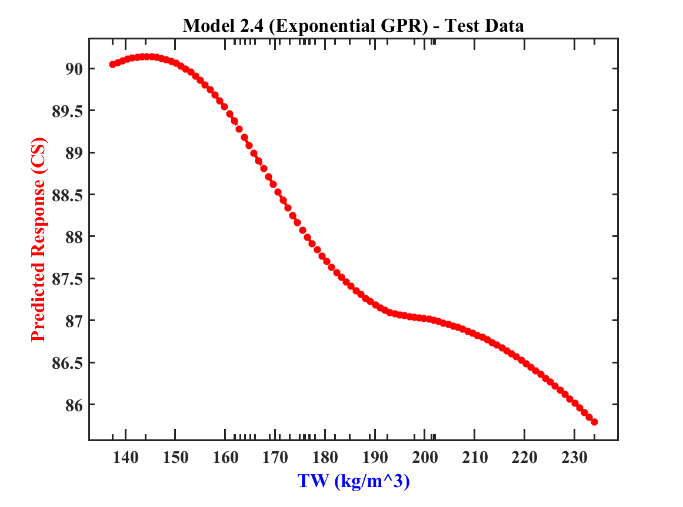

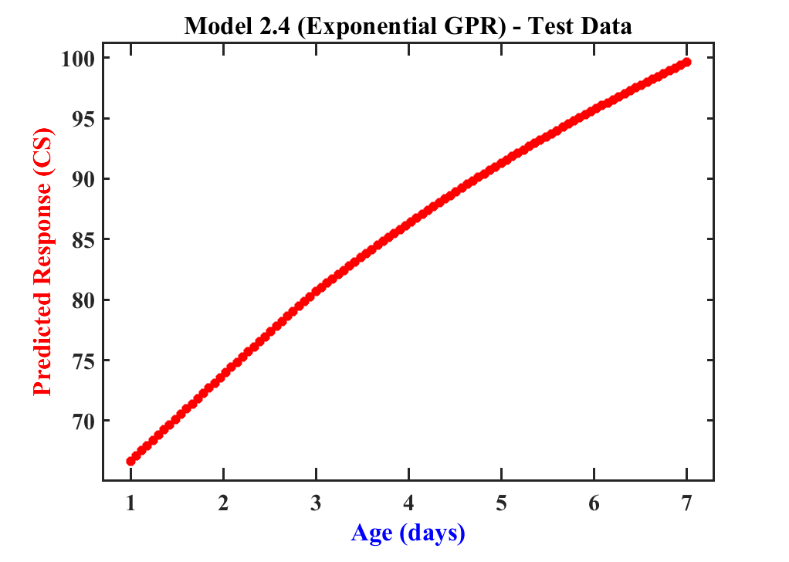


g) h)


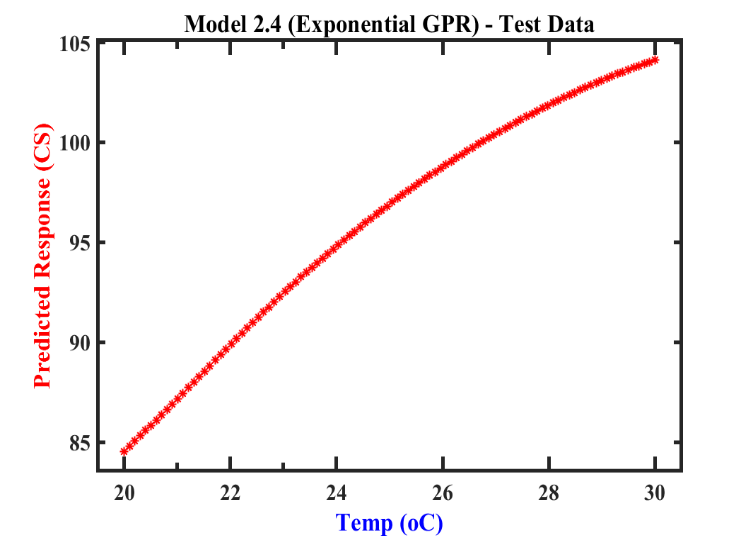


i)

Fig. S2**.** PDP analysis of the effects of independent variables on CS.
